# Isolation of Human Islets for Autologous Islet Transplantation in Children and Adolescents with Chronic Pancreatitis

**DOI:** 10.1155/2012/642787

**Published:** 2012-02-08

**Authors:** Rita Bottino, Suzanne Bertera, Maria Grupillo, Patricia R. Melvin, Abhinav Humar, George Mazariegos, A. James Moser, R. Matthew Walsh, John Fung, Andres Gelrud, Adam Slivka, Kyle Soltys, Martin Wijkstrom, Massimo Trucco

**Affiliations:** ^1^Division of Immunogenetics, Department of Pediatrics, Rangos Research Center, Children's Hospital of Pittsburgh, 4401 Penn Avenue, Pittsburgh, PA 15224, USA; ^2^RiMeD Foundation, via Bandiera 11, Palermo 90133, Italy; ^3^Department of Surgery, School of Medicine, University of Pittsburgh, 200 Lothrop Street, Pittsburgh, PA 15213, USA; ^4^Thomas Starzl Transplantation Institute, School of Medicine, University of Pittsburgh, E1540 Biomedical Science Tower (BST) 200 Lothrop Street, Pittsburgh, PA 15261, USA; ^5^Department of Surgery, Children's Hospital of Pittsburgh, and School of Medicine, University of Pittsburgh, 4401 Penn Avenue, Pittsburgh, PA 15224, USA; ^6^Division of Surgical Oncology, Department of Surgery, University of Pittsburgh, 5150 Center Avenue, Pittsburgh, PA 15232, USA; ^7^Department of Hepato-Pancreato-Biliary and Transplant Surgery, Digestive Disease Institute, Cleveland Clinic, 9500 Euclid Avenue, Cleveland, OH 44195, USA; ^8^Division of Gastroenterology, Department of Medicine, University of Pittsburgh Medical Center, 200 Lothrop Street, Pittsburgh, PA 15213, USA

## Abstract

Chronic pancreatitis is an inflammatory disease of the pancreas that causes permanent changes in the function and structure of the pancreas. It is most commonly a complication of cystic fibrosis or due to a genetic predisposition. Chronic pancreatitis generally presents symptomatically as recurrent abdominal pain, which becomes persistent over time. The pain eventually becomes disabling. Once specific medical treatments and endoscopic interventions are no longer efficacious, total pancreatectomy is the alternative of choice for helping the patient achieve pain control. While daily administrations of digestive enzymes cannot be avoided, insulin-dependent diabetes can be prevented by transplanting the isolated pancreatic islets back to the patient. The greater the number of islets infused, the greater the chance to prevent or at least control the effects of surgical diabetes. We present here a technical approach for the isolation and preservation of the islets proven to be efficient to obtain high numbers of islets, favoring the successful treatment of young patients.

## 1. Introduction

Pancreatitis is an inflammatory disorder of the pancreas that can be acute or chronic. Acute pancreatitis is an “event” whereas chronic pancreatitis is a “process” [1]. Acute pancreatitis occurs suddenly and resolves without significant irreversible damage to the gland. Chronic pancreatitis (CP) can be considered the result of repeated acute inflammatory events of varying duration. The long-standing inflammatory injuries produce chronic inflammatory infiltrates, loss of normal pancreatic cells and fibrosis. In children, environmental factors seem to play a smaller role in the etiology of chronic pancreatitis than found in adults [2]. A large percentage of children with CP are still considered to have idiopathic disease. A significant fraction has congenital anomalies of the biliary tree, pancreas, stomach, or duodenum. More than half of children with CP have mutations in the genes encoding the cystic fibrosis transmembrane conductance regulator (CFTR), cationic trypsinogen (PRSS1) or serine protease inhibitor Kazal type 1 (SPINK1) [3–5]. Mutations in CFTR and SPINK1 produce sporadic disease, whereas mutations in PRSS1 result in autosomal dominant hereditary pancreatitis [1]. Mutations in SPINK1 increase the risk of chronic pancreatitis, and are considered disease modifiers. Specific CFTR genotypes are significantly associated with pancreatitis but the pathogenesis is complex, and other genes likely modify the risk [6]. For instance, the combination of mutations in CFTR and SPINK1 increases the risk of CP to around 900-fold, much higher than the risk of a mutation in either gene alone [1].

The most evident symptom of CP is recurrent or chronic abdominal pain. This pain can be so debilitating that children are unable to attend school or perform any normal activity due to frequent hospitalizations. Growing up under such conditions may lead to depression, and the combination of the pain and depression frequently results in a dependence on narcotic analgesics [7, 8]. Once medical treatments are no longer efficacious, the only possible solution is surgical treatment. Surgical options to treat CP depend on etiology and the morphologic consequences of the disease. Some patients are candidates for endoscopic pancreatic ductal drainage, and others for resection treating a focal disease. Total pancreatectomy is reserved for diffuse changes, where no other surgical options are reasonable. Besides the life-long need for substituting the exocrine function of the pancreas, the endocrine function is also lost.

To reduce the severe consequences of the complete removal of the pancreas and to save part of the endocrine function at least for a time, the isolated islets of Langerhans can be returned to the patient by injecting them into the portal vein, so that they will eventually make their “home” in the sinusoids of the liver [9, 10]. Isolation of islets from the pancreas of young individuals is technically challenging even when dealing with healthy donor organs. The concern of producing an adequate yield of islets from pancreata affected by CP has been one of the limiting factors in undertaking this procedure [11].

Our center has developed a successful method for the isolation of islets from juvenile pancreatic organs derived from deceased organ donors [12] and has applied it, with some modifications, to pancreata excised from CP patients to allow autologous islet transplantation.

Hereby, we describe the technical approach and the results of the isolation of islets from the first 10 young CP patients who also received them back as autotransplants.

## 2. Materials and Methods

### 2.1. Islet Isolation

Total pancreaticoduodenectomy has been performed with subsequent islet processing from pediatric patients with CP at the Children's Hospital of Pittsburgh of UPMC and the Cleveland Clinic since 2009 (Table 1). Once harvested, pancreata were immediately transferred in cold preservation fluid (HTK) to our laboratory with cold ischemia time ranging between 30 minutes and 4 hours.

The method for islet isolation is a modification of the one that our group developed [13] and further adapted to pediatric pancreata [12]. Briefly, after the removal of fat, connective tissue, and blood vessels, the pancreas is washed in a cocktail of antibiotics, weighed and cut at the level of the neck to access the pancreatic duct. Catheters are placed in both sides of the transected duct, one catheter towards the head, and the second towards the body, both secured with suture. The diameter of the pancreatic ducts can vary; however, in pancreata with CP, it is commonly dilated. An appropriate catheter size is required to prevent the leakage of digestive enzyme solution; usually it is never smaller than 16–18 G in diameter. A blend of exogenous enzymes including collagenases and neutral proteases (Serva, GMP grade, Heidelberg, Germany), freshly dissolved in HBSS (Mediatech-Cellgro, Inc., Manassas VA, USA), is prewarmed to 28–30°C and introduced intraductally in a special apparatus that Dr. Ray Rajotte (University of Alberta) designed to allow for controlled continuous infusion of enzymes in the pancreas at given temperatures. Infusion of enzymes usually distends the pancreas and increases its volume quite substantially; however, in CP, due to the intrinsic features and fibrosis, often only a limited distension is obtained. The pancreatic organ is then transferred to the Ricordi digestion chamber where a solution containing the same enzyme blend circulates, while shaking is performed to produce the mechanical disruption of the pancreatic tissue [14].

In order to monitor the effective digestion of the pancreatic tissue, samples are obtained from the chamber and monitored every 2–4 minutes. To analyze them, the samples are suspended in a solution of HBSS containing Dithizone (DTZ), a dye that colors the islets in red to distinguish them from unlabeled exocrine and ductal tissue [15]. When islets free of exocrine tissue are visible, the circuit is then opened, and the tissue is collected in conical tubes. As described previously [13], our group has developed a method that allows, on one hand, to remove the islets from the circuit as soon as they are free and, on the other, to reinsert the enzymes back into the circuit itself to continue the digestion of yet undisrupted tissue.

Once the digestion is completed and the isolated islets and pancreatic cells are obtained, several washes in cold RPMI supplemented with human serum albumin (2.5% total volume) are carried out. The cell pellet size is measured, and a decision is made whether to use a cell separator (Cobe 2991, Gambro BCT, Lakehood, CO, USA) to purify and concentrate the islets, or to begin preparing the cells for infusion. In adults, the pellet size cutoff for further purification is 15 mL. For pediatric patients, we follow the guidelines defined by the Minnesota group [16] that suggest a maximum of 0.2 mL/Kg body weight but never more than the 15 mL limit. This value dictates the choice for the purification step. Islets are delivered to the patient by slow infusion into the portal vein resuspended in infusion medium: CMRL 1066 medium (Mediatech) supplemented with human serum albumin (5%), heparin (10 u/Kg recipient body weight), and ciprofloxacin (2 *μ*g/mL). Each 5 mL of cell pellet is resuspended in 200 mL of infusion medium, which makes up the content of one infusion bag (blood transfusion bags, Fenwal, Lake Zurich, IL, USA). Up to three bags, corresponding to 600 mL and a maximum of 15 mL of cell pellet may be infused.

### 2.2. Islet Quantification and Quality Control Testing

Islet yield is determined by direct count of the islets stained with DTZ, converting the different islet sizes into islet equivalent numbers (IEQ) [14]. An islet equivalent is equal to the volume of an islet with 150 *μ*m diameter. Viability is evaluated prior to islet transplantation by trypan blue exclusion assay or by a dual fluorescence method with calcein AM and propidium iodide [17]. Samples of the islet cells as well as of the procurement fluid (the fluid in which the pancreas is preserved until isolation) are analyzed by Gram stain, and sterility culture for microbiological contaminations. Islet samples are also analyzed for mycoplasma and endotoxin content using FDA-approved methods. As a parameter of islet function after transplantation, fasting serum C-peptide levels were measured using a standard chemiluminescence method.

## 3. Results

### 3.1. Patient Age, Body Weight, and Pancreas Size

Table 1 summarizes the age of the patients, their body weight at the time of pancreatectomy and islet autotransplantation, and the pancreatic organ weight. In all cases, pylorus-preserving total pancreaticoduodenectomy was performed, and the head, body, and tail of the pancreas were all sent to the islet lab as sources of islet cells after back-table flushing (HTK solution) of the splenic and gastroduodenal arteries.

### 3.2. Islet Yield

**Figure 1:**
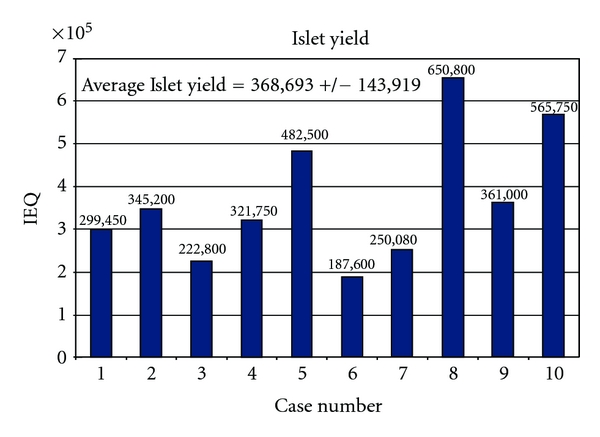
Islet yield expressed as islet equivalent numbers.

**Figure 2:**
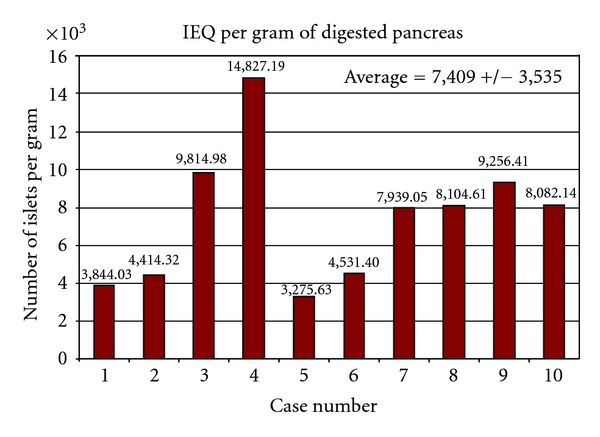
Islet yield expressed as islet equivalent numbers per gram of pancreatic tissue actually digested.

**Figure 3:**
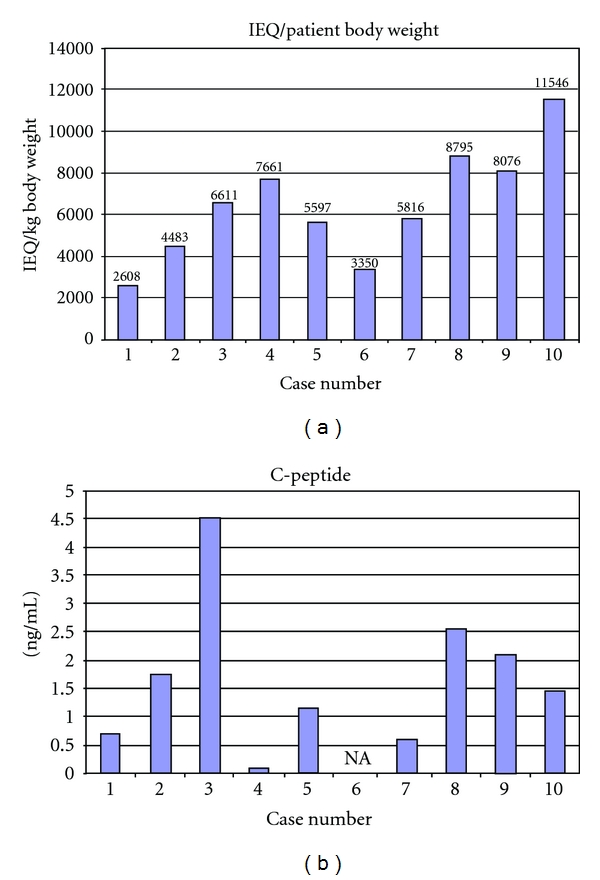
In (a) Islet yield expressed as islet equivalent (IEQ) numbers per kg of patient body weight. In (b) Serum C-peptide levels 6 months after transplant. Case no. 9: Serum C-peptide levels 3 months post-transplant. NA: not available.

Islet yield is expressed as the total islet number (IEQ) per pancreas (Figure 1), islet number (IEQ)/weight of digested pancreas (Figure 2), and islet number (IEQ)/Kg body weight of the recipient (Figure 3(a)). From a technical standpoint, IEQ is expressed per gram of pancreas digested, and the density of the islets is indicative of the efficiency of the extraction procedure. However, for clinical purposes, the most indicative value is IEQ/Kg body weight. Studies have shown that infusion of at least 2500 IEQ/Kg is associated with an increased chance of achieving satisfactory blood glucose control, while infusion of ≥5000 IEQ/Kg is very predictive of good metabolic function and even insulin independence [18–21].  Figure 3(b) shows posttransplantation serum C-peptide levels relative to this cohort of patients. The threshold of 2500 IEQ/kg for achieving satisfactory blood glucose control in recipients of autologous islet transplantation [18] seems to apply also in our cases, with the only exception of case no. 4. That is the single case in which a sufficiently high C-peptide level was not obtained even with IEQ/kg greater than 7000.

### 3.3. Technical Management of the Isolation

**Figure 4:**
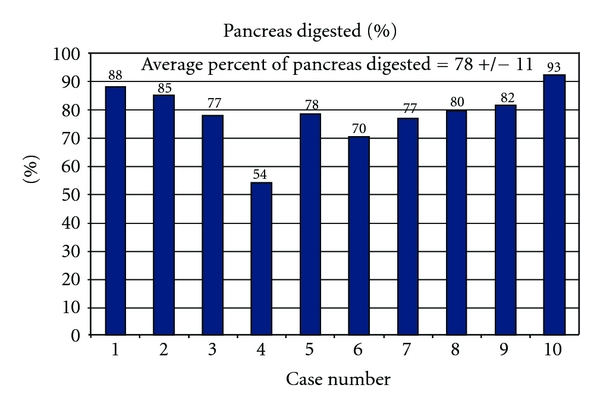
At the end of the isolation, some tissue is left in the digestion chamber. Of the undigested portion, only less than 20% was glandular, with a prevalence of fibrotic, calcified, and ductal tissue.

The aim of the isolation procedure is the recovery of the greatest number of islets possible from the pancreatic organ; thus, care must be taken to assure enough time for pancreatic digestion to maximize islet yield, while at the same time not losing islets to enzymatic or mechanical overdigestion. It is therefore essential that the pancreatic organ is optimally perfused with exogenous enzymes by intraductal injection of a solution containing collagenases and neutral proteases. In all cases, the pancreatic duct is located and cannulated using catheters of appropriate size to fit snugly in the duct (14 to 24 G in size). Sometimes in CP cases, the presence of calcifications renders the distribution of the enzyme in the pancreatic parenchyma particularly difficult. Consequently, distension of the organ is less evident in pancreata with pancreatitis in comparison with organs from healthy patients. In all of our cases, however, a substantial increase in pancreatic volume was achieved. This is indicative of good perfusion. At the end of the isolation (enzymatic and mechanical) the tissue left in the digestion chamber is weighed, and then by visual inspection it is determined how much of what remains is pancreatic tissue (i.e., islet containing tissue) and how much is nonpancreatic, such as ductal tissue, fibrotic tissue, and blood vessels. The amount of nonpancreatic tissue is not important for our purposes since islets cannot be obtained from it, but the less undigested pancreatic tissue found, the more efficient we consider the digestion process.  Figure 4 indicates the percent of tissue left undigested from each organ treated. Our data indicates that more than 70% of the initial tissue was digested, and of the remaining undigested tissue just under 25% was estimated to be glandular (i.e., not ductal, connective, or fibrotic tissue). Considering that CP substantially affects the anatomy of the pancreas, these results are rather encouraging. In fact, they are not significantly different from the data obtained in isolations of organs from healthy donors (data not shown).

### 3.4. Digestion Management

**Figure 5:**
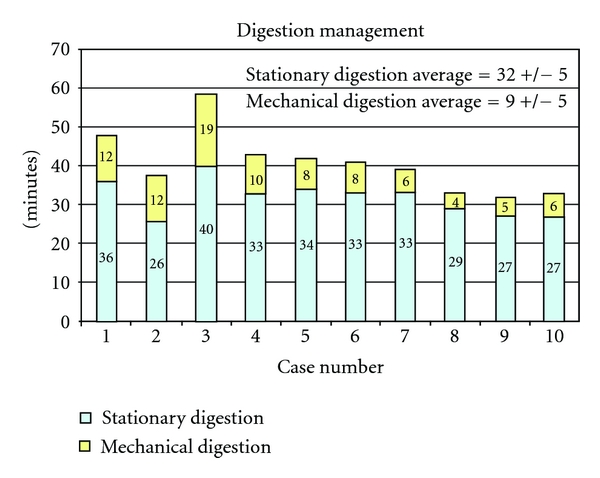
The pancreatic organ of young patients is subjected first to a stationary digestion characterized by prolonged infusion of prewarmed collagenase-neutral protease solution. Mechanical shaking is carried out after stationary digestion and limited in time to avoid excessive breakage of islets embedded in exocrine tissue.

A combination of stationary and mechanical digestions is usually necessary for efficient pancreatic digestion. Stationary digestion is defined as the time in which the pancreas is perfused with collagenases in the Rajotte chamber. Mechanical digestion is considered to begin when the pancreas is transferred into the Ricordi chamber to the point in which the shaking, added to break down the extracellular matrix, is stopped. In younger donors, we have achieved better results by prolonging the stationary digestion.  Figure 5 shows the relative proportion of stationary over mechanical digestion. As also described by Balamurugan et al. [13], longer stationary digestion in younger donors is associated with a reduced yield of mantled islets (i.e., nonendocrine cells surrounding the islets). While a small mantle surrounding the islets may not be considered detrimental and actually may confer protection [22], there is general agreement that a thick mantle may prevent nutrients from efficiently reaching the islet core, contributing to necrosis and islet cell apoptosis.

### 3.5. Final Product

**Figure 6:**
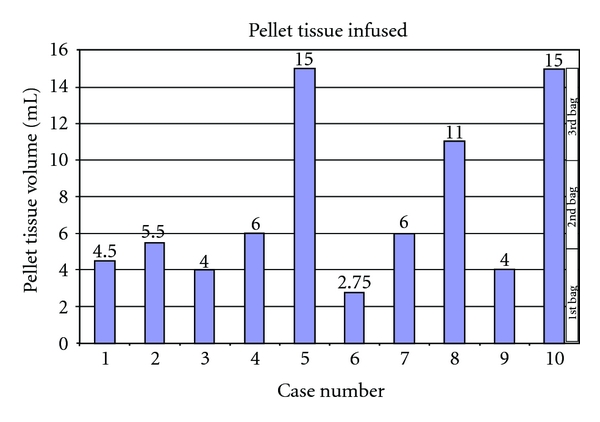
Amount of tissue infused. After digestion and purification (when applied), the pancreatic cell pellet (comprised between 2.75 and 15 mL) is suspended in medium containing human serum albumin and bagged for infusion. Each bag contains a maximum of 5 mL of pellet tissue.

In our hands, the isolation yielded a cell pellet that varied between 3 and 45 mL in volume and composed mainly of acinar cells and isolated islets. In most cases, the cell pellet has a positive correlation with the weight of the pancreas. Since a maximum of 15 mL of cell pellet is desirable for infusion (and even less in children smaller than 75 Kg), any cell pellet higher in volume requires purification to concentrate the islets in a lower volume by reducing the amount of exocrine tissue. Purification was carried out in three cases: case 1, 5, and 8. The cell pellet was reduced from 20 mL to 4.5 mL, from 45 mL to 15 mL, and from 21 mL to 11 mL, respectively.  Figure 6 indicates how much cell pellet was obtained and infused and how many infusion bags were delivered. Each bag can contain no more than 5 mL of cell pellet in 200 mL infusion solution. In the cases in which purification was carried out, pellet size was efficiently reduced to the standard volume for injection. It was estimated that less than 15% of the final islet mass was lost due to the purification process.

### 3.6. Microbiological Contamination

Samples of procurement fluid and islet cells taken to check sterility are commonly found positive for bacterial contamination. The presence of bacteria is probably secondary to the underlying disease, previous endoscopic procedures and, more importantly, to the presence of the duodenum with the specimen. Given the close relationship of the pancreas with the duodenum, and the typical defective drainage of the pancreatic juices, contamination from intestinal flora is often found in the procurement fluid as well as the islets. Similar incidence of bacterial contamination was also reported by others [23].

**Figure 7:**
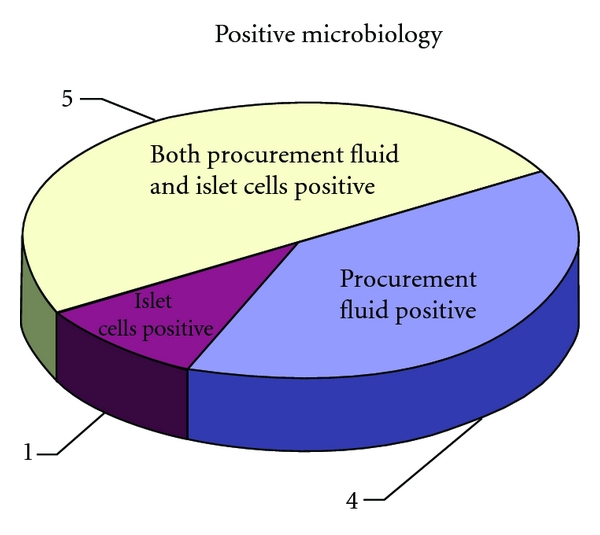
Number of cases in which the pancreas procurement fluid and/or the islet cells showed positive microbiology.

Figure 7 and Table 2 show the results of the sterility analysis and the isolated bacteria. Procurement fluid was quite commonly positive. In some cases, the isolate from the procurement fluid was also found within the islets. However, in some few cases, the islets had different isolates. Antibiotic therapy has been effective in controlling possible effects of the contaminations *in vivo*, and, in our experience, no cases of postoperative infections were identified.

Mycoplasma and endotoxin levels are shown in Table 3.

## 4. Discussion

Chronic pancreatitis represents a very debilitating and serious disorder, particularly in juveniles. The decision to perform a total pancreatectomy is a weighty one considering both short-term and long-term morbidity. Although it is up to the gastroenterologist's expertise to treat this disease and to decide when all the available routes of intervention have been exhausted, once surgical removal of the pancreas becomes an option, it is important to consider islet cell autotransplantation to reduce or eliminate a major source of morbidity, diabetes. The surgical intervention is unchanged whether autoislets are reinfused, regardless of the potential residual endocrine function. If the islets are not given back to the patient, they will be discarded. Rather than not using the islets, even minimal endocrine function returned to the patient, ethically and clinically justifies the procedure of islet isolation and autotransplantation [24]. We believe that any additional risk, which is generally minimal to the patient would be more than outweighed by the benefit, even considering limitations such as the quality and quantity of the available islets, deteriorated as they are after the prolonged inflammation of the organ, and the less than ideal location of the infusion into the liver. Even if the islets available for transplantation are limited in mass, it is proven that a limited quantity of physiologically generated insulin can be of great advantage for the patient. Administrations of recombinant insulin could be completely avoided or the quantity reduced.

Although the variables that clinically influence the efficiency of each transplant are high in numbers so that a direct relationship between IEQ/kg and C-peptide production is difficult to demonstrate, we can agree that the previously determined threshold of 2500–3000 IEQ/kg is associated with most successful clinical results [18].

 From the islet allotransplantation setting, we also learned that even minimal quantities of insulin and C-peptide can reduce, respectively, hypoglycemia unawareness and its frequently tragic consequences, as well as the blood vessel atherosclerotic deterioration, principal cause of all typical diabetes complications [25, 26]. Also, because it is an autotransplant, the absence of a need for immunosuppression allows not only for a better engraftment of the islets, but also for a potentially longer graft survival time than is observed for the islets used in allotransplantation. The improved survival is due in part to the absence of rejection, and in part to the fact that the most used immunosuppressive regimens include drugs that are toxic for the insulin-producing beta cells [27].

The frequently less than lifelong survival of the graft might not be due entirely to the stress that the isolation protocol itself imposes on the islets, but also to the less than ideal site of the infusion. The sinusoids of the liver, while optimal to guarantee blood exposure to the islets and a proper geographical location—being a part of the portal system in which insulin is physiologically secreted—might not be the most suited site for properly lodging them. It is demonstrated that the islets transplanted into the liver accumulate amyloid deposits that, not only damage the liver's structure [28, 29], but also tends to physically isolate the transplanted tissue, reducing its ability to obtain sufficient oxygen and nutrients. While looking for more suitable sites [30, 31], the present possibility of providing some diabetes-free years or at least years of reduced insulin needs, still supports the procedure of islet autotransplantation.

If, in many cases, the characteristics of the pancreas are such that they do not allow for the harvest of a sufficiently large mass of islets to completely satisfy the recipient's insulin needs, a very sizeable improvement has been obtained adapting our isolation procedure to better digest the pancreas damaged by CP. We have found that by optimizing the perfusion of collagenases through the entire pancreas, by a technique in which we clamp any leaks, allows for a more complete digestion. In addition, a longer period of this stationary digestion is particularly beneficial to younger patients in producing a larger yield of islets. Another hallmark of our process is the flexibility in the management of the digestion, tailoring the amount of stationary and mechanical digestion based on direct monitoring of the islets released under the microscope rather than rigidly following a fixed standard of timing. The number of successful islet autotransplants in young patients since we started this protocol certainly supports our positive conclusions and leads us to continue to search for alternative ways to better digest the organ, better protect the islets during the isolation, and find a more suitable site to transplant the isolated islets.

We are currently testing drugs able to reduce the generation of dangerous reactive oxygen radicals during the organ perfusion prior to islet isolation, during the isolation itself, and even after transplantation, infusing the drug with the islets and into the recipient intravenously once the transplant is completed [32, 33]. Better sites for allo- and possibly autotransplants are also being investigated, such as the gastric submucosa, which is accessible endoscopically [34]. Through the same noninvasive route, we can also easily obtain biopsies useful to monitor the survival of the graft.

Some CP patients, because of family history or genetic mutations, have a higher risk for developing pancreatic cancer. A pancreatectomy will, of course, remove this risk. However, the question remains if it is safe to isolate and return the islets to these patients. Research into this question is still relatively new; however, it appears that in these cases an autotransplant of islets into the liver does not increase the risk of the patient developing cancer [35]. The death of the majority of the acinar cells during the isolation procedure and the reduced quantity of ductal material remaining after isolation may explain these positive results.

Autotransplantation has been demonstrated from the results we have produced to date to be a procedure that is worth continuing while investigating additional methods to improve outcome.

## 5. Conclusions

Total pancreatectomy is the last resort for chronic pancreatitis. This treatment leads to surgical diabetes. The decision to perform this drastic but sometimes only effective method of treatment is especially difficult when dealing with adolescents who face a lifetime of care for diabetes and its complications.

Autotransplantation of pancreatic islets has shown the ability to restore insulin function but was not considered to be an effective treatment for adolescents due to the difficulty in producing enough islets. Our experience, however, has shown that we are able to obtain a sufficient number of islets that when transplanted back into patients even under the age of 18, confers benefits to their health and the chance to achieve insulin independence.

## Figures and Tables

**Table 1:** Patient age—patient body weight—pancreas weight.

Case no.	Patient age (Yr)	Patient Wt. (Kg)	Pancreas Wt. (g)
1	18	115	89
2	18	77	92
3	9	34	29
4	11	42	40
5	15	86	189
6	16	56	59
7	18	43	41
8	15	74	101
9	15	45	48
10	10	49	76

**Table 2:** Germ identification.

Case no.	Procurement fluid	Islet cells
1	Coagulase-negative staph no. 1, 2, 3	None
2	None	Coagulase-negative staph
3	Coagulase-negative staph	Viridans Streptococcus
4	Enterobacter cloacae	Enterobacter cloacae
	Klebsiella pneumoniae	Klebsiella pneumoniae
5	Enterobacter cloacae No. 1, 2	Enterobacter cloacae No. 1, 2
		Viridans Streptococci
6	Escherichia coli	Klebsiella oxytoca
7	Enterococcus faecium	Enterococcus faecium
8	Micrococcus species	None
	Diphtheroids	
9	Diphtheroids	None
	Klebsiella Pneumoniae	
10	Peptostreptococcus Species	None
	Diphtheroids	

**Table 3:** Endotoxins (EIU)/patient body Wt. (Kg), mycoplasma results.

Case no.	1	2	3	4	5	6	7	8	9	10
Endotoxins/patient Wt.	0.63	N/A	5.88	N/A	3.48	0.54	0.70	0.88	0.67	1.22
Mycoplasma	Neg.	Neg.	Neg.	Neg.	Neg.	Neg.	Neg.	Neg.	Neg.	Neg.
